# Diffuse Alveolar Hemorrhage Associated with Warfarin Therapy

**DOI:** 10.1155/2015/350532

**Published:** 2015-08-12

**Authors:** Bülent Kaya, Ibrahim Yildiz, Reshat Mehmet Baha, Neslihan Ebru Eryaşar Zeytun, Azize Yetisgen

**Affiliations:** ^1^Nephrology Department, Osmaniye State Hospital, Turkey; ^2^Cardiology Department, Osmaniye State Hospital, Turkey; ^3^Infectious Diseases Department, Osmaniye State Hospital, Turkey

## Abstract

Diffuse alveolar hemorrhage (DAH) is a life-threatening clinical pathologic syndrome caused by a variety of diseases. We report a case of DAH related to therapy of warfarin use. In this case report, we present the diffuse alveolar hemorrhage case as a rare and life-threatening complication of warfarin.

## 1. Introduction

Diffuse alveolar hemorrhage (DAH) is a life-threatening clinical pathologic syndrome caused by a variety of diseases. We report a case of DAH related to therapy of warfarin use. DAH due to warfarin therapy has been rarely reported in the literature. In this paper, we describe the case of a 59-year-old man with a history of mitral valve replacement (MVR) hypertension (HT), diabetes mellitus (DM), and chronic renal failure (CRF) treated with warfarin, who was admitted to our institution with haemoptysis and dyspnea. Alveolar hemorrhage was suspected clinically and radiologic findings were subsequently confirmed by bronchoscopy. The patient was successfully treated with 6 units of fresh frozen plasma (FFP), 4 units of red blood cell (RBC) transfusion, and hemodialysis.

## 2. Case Presentation

A 59-year-old man with a past medical history of HT, DM, MVR, and CRF was admitted to our institution complaining of haemoptysis and shortness of breath over the previous 24 h.

On examination, he was noted to be in mild respiratory distress and was coughing up blood-tinged sputum. His vital signs were temperature 37,4°C, pulse 106 bpm, and regular, respiratory rate 20 per minute, blood pressure 140/77 mm Hg, and oxygen saturation 94% on room air. He had rhythmic metallic first heart sound and a normal second heart sound. He also had diffuse wet crackles over both lung fields. The rest of his physical examination was unremarkable.

The patient's medications on the day of admission were amlodipine 10 mg PO daily, warfarin 2,5 mg PO daily, and carvedilol 12,5 mg PO daily.

The patient's laboratory findings were WBC 8,8 haemoglobin 7,5 g/dL, haematocrit 22,3%, platelets 148,000, INR 7,9, PT 94 s, a PTT 120 s, random glucose 177 mg/dL, serum sodium 141 mmol/L, serum potassium 4 mmol/L, and serum creatinine 8,6 mg/dL. Urinalysis showed very high number of RBCs without RBC casts. The rest of the laboratory results, including D-dimer level and cardiac enzymes, were unremarkable.

The patient was started empirically on moxifloxacin for possible community acquired pneumonia. Serum levels of C-ANCA, P-ANCA, ANA, and anti-ds-DNA were negative. Transthoracic echocardiography showed left ventricular ejection fraction of 0.45 and a normal mechanical mitral valve prosthesis with normal gradient. Abdominal ultrasound showed bilateral normal kidneys and bilaterally increased renal cortical echogenicity.

**Figure 1:**
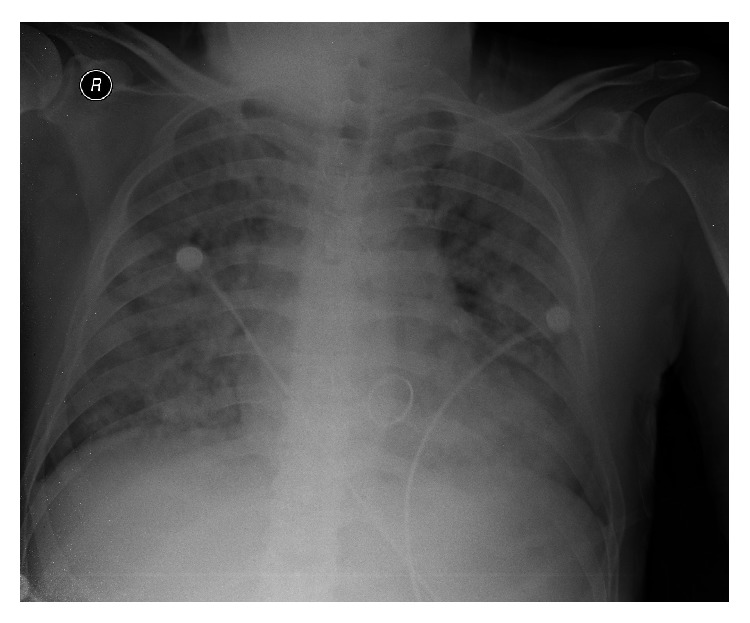
Chest X-ray on admission. Chest X-ray showed alveolar opacities in both lungs, especially in the perihilar and pericardiac zones.

**Figure 2:**
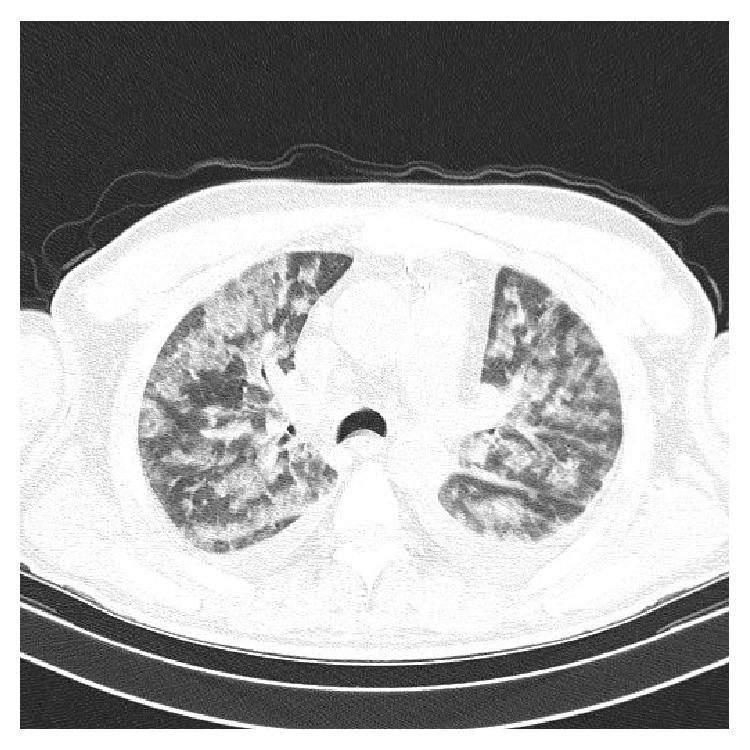
Computed tomography of the chest on admission. High resolution CT of the chest showed bilateral patchy airspace disease.

Chest radiographs revealed that the lungs were bilaterally infiltrated (Figure 1). A high resolution computed tomographic study of the thorax disclosed DAH (Figure 2), the presence of which was proved by bronchoscopy.

The patient was a known case of CFR due to diabetic nephropathy for the last five years. A double lumen haemodialysis catheter was inserted in his right internal jugular vein as a vascular access for emergency haemodialysis. In total, 8 hemodialysis sessions were carried out during hospital stay. FFP and RBC transfusions were given during hemodialysis sessions to avoid hypervolemia. After FFP administration, coagulopathy improved gradually. When the INR fell below 2, a low molecular weight heparin was started. End-stage renal failure due to diabetic nephropathy was assumed for the patient and he was discharged upon a chronic hemodialysis program (Table 1).

## 3. Discussion

DAH is a rare occurrence in clinical practice. It can be manifestation of a variety of diseases. The disorders that can cause alveolar hemorrhage involve autoimmune diseases (Wegener granulomatosis, Good Pasteur's syndrome, systemic lupus erythematosus, antiphospholipid antibody syndrome, and Behçet's syndrome), pulmonary infections, cardiac disorders (mitral stenosis), coagulation disorders, bone marrow/solid organ transplantation, toxic exposures, drug reactions (amiodarone, methotrexate, etc.), and idiopathic pulmonary haemosiderosis [1]. A few drugs, including the widely used anticoagulant warfarin, have been documented to cause this potentially lethal condition [2, 3].

Warfarin is a vitamin K antagonist anticoagulant drug and has potential adverse effects. A vitamin K antagonist (VKA; e.g., warfarin) is recommended in selected patients with metallic prosthetic heart valves to prevent valve thrombosis and thromboembolic events. Warfarin dosing is guided by the use of the international normalized ratio (INR). In patients with a mechanical mitral heart valve, VKA therapy with a target of 3.0 (range 2.5 to 3.5) is suggested [4].

Unfortunately, many factors such as diet, concurrent medication changes, poor compliance, or alcohol consumption may result in unexpected fluctuations in INR levels. The main complication of oral warfarin therapy is bleeding, especially from vital organs such as the brain or lung [5]. Alveolar hemorrhage is difficult to diagnose and has high mortality if the treatment is not being started as soon as possible.

We propose that the patients on anticoagulation therapy require strict monitoring with PT/INR to avoid serious bleeding complications related to overanticoagulation.

## Figures and Tables

**Table 1:** The patient's labarotary parameters, according to date and levels.

Date	10.1.15	11.1.15	12.1.15	14.1.15	15.1.15	16.1.15	17.1.15	19.1.15	21.1.15	22.1.15	23.1.15	24.1.15

INR	7,88	4,63	2,36	2,31	1,69	1,59	1,31	1,3	1,18	1,15	1,1	1,1

PTZ	94,2	49,3	25,8	25,3	18,7	17,7	14,7	14,5	13,2	13,1	12,6	11,8

APTT	120	82,4	60,8	48,4	40,3	29,4	30,4	36,9	31,4	30,2	28,5	28,2

Creatinin (mg/dL)	10,8	8,6	7,1	5	3,8	4,9	7,2	6	4,1	5,2	7,1	6

Hgb (g/dL)	8	9,3	8,4	7,5	9,8	9,6	9,45	9,6	9,8	−	9,6	−

Haemodialysis (Session)	+	+	+	+	−	−	+	+	−	−	+	+

FFP (unit)	++	++	+	+	−	−	−	−	−	−	−	−

RBC (unit)	+	−	+	++	−	−	−	−	−	−	−	−
